# Iterative reconstruction incorporating background correction improves quantification of [^18^F]-NaF PET/CT images of patients with abdominal aortic aneurysm

**DOI:** 10.1007/s12350-019-01940-4

**Published:** 2019-11-11

**Authors:** Mercy I. Akerele, Nicolas A. Karakatsanis, Rachael O. Forsythe, Marc R. Dweck, Maaz Syed, Robert G. Aykroyd, Steven Sourbron, David E. Newby, Charalampos Tsoumpas

**Affiliations:** 1 Biomedical Imaging Science Department, Leeds Institute of Cardiovascular and Metabolic Medicine, University of Leeds, Leeds, LS2 9NL UK; 2 Division of Radiopharmaceutical Sciences, Department of Radiology, Weil Cornell Medical College of Cornell University, New York, NY USA; 3 British Heart Foundation Centre for Cardiovascular Science, University of Edinburgh, Edinburgh, UK; 4 Edinburgh Imaging Facility, Queen’s Medical Research Institute, University of Edinburgh, Edinburgh, UK; 5 Department of Statistics, University of Leeds, Leeds, UK

**Keywords:** Abdominal aortic aneurysm, positron emission tomography, spill in, background correction

## Abstract

**Background:**

A confounding issue in [^18^F]-NaF PET/CT imaging of abdominal aortic aneurysms (AAA) is the spill in contamination from the bone into the aneurysm. This study investigates and corrects for this spill in contamination using the background correction (BC) technique without the need to manually exclude the part of the AAA region close to the bone.

**Methods:**

Seventy-two (72) datasets of patients with AAA were reconstructed with the standard ordered subset expectation maximization (OSEM) algorithm incorporating point spread function (PSF) modelling. The spill in effect in the aneurysm was investigated using two target regions of interest (ROIs): one covering the entire aneurysm (AAA), and the other covering the aneurysm but excluding the part close to the bone (AAA_exc_). ROI analysis was performed by comparing the maximum SUV in the target ROI (SUV_max_(T)), the corrected cSUV_max_ (SUV_max_(T) − SUV_mean_(B)) and the target-to-blood ratio (TBR = SUV_max_(T)/SUV_mean_(B)) with respect to the mean SUV in the right atrium region.

**Results:**

There is a statistically significant higher [^18^F]-NaF uptake in the aneurysm than normal aorta and this is not correlated with the aneurysm size. There is also a significant difference in aneurysm uptake for OSEM and OSEM + PSF (but not OSEM + PSF + BC) when quantifying with AAA and AAA_exc_ due to the spill in from the bone. This spill in effect depends on proximity of the aneurysms to the bone as close aneurysms suffer more from spill in than farther ones.

**Conclusion:**

The background correction (OSEM + PSF + BC) technique provided more robust AAA quantitative assessments regardless of the AAA ROI delineation method, and thus it can be considered as an effective spill in correction method for [^18^F]-NaF AAA studies.

**Electronic supplementary material:**

The online version of this article (10.1007/s12350-019-01940-4) contains supplementary material, which is available to authorized users.

## Introduction

Abdominal aortic aneurysm (AAA) is the irreversible dilation of the abdominal aorta to greater than 30 mm diameter, representing a more than 50% increase compared with a normal aortic diameter. As the disease progresses, the aorta becomes more enlarged, and could potentially rupture unless there is a timely clinical intervention.^1^ AAA rupture is life-threatening, with more than 80% mortality rate and accounts for over 8000 deaths annually in the UK.^2^ The exact causes of the emergence and progression of AAA are not completely understood, however, the most common risk factors for AAA development are smoking, male sex, hypertension and advancing age.^3^,^4^ In clinical practice, once AAA is identified, the patient enters a surveillance programme, with serial measurements of the aortic diameter (commonly using ultrasound) until the aneurysm meets a ‘diameter threshold’ for considering intervention (typically 55 mm). However, the use of the aortic diameter alone as a prognostic measure is somewhat limited because aneurysms vary in their progression rate and risk of rupture.^5^–^7^ This suggests the need for more reliable tools to identify patients at risk of AAA expansion and rupture, and so the use of molecular imaging biomarkers to assess the biological activity of AAA is a field of increasing interest.

At the moment, [^18^F]-FDG is the most commonly used radiotracer for positron emission tomography (PET) imaging of AAA due to its property of detecting vascular diseases caused by inflammation,^8^,^9^ which is a key process in AAA progression.^10^ Past studies have shown no correlation between [^18^F]-FDG PET uptake and aneurysm diameter.^11^–^13^ It was then concluded that PET uptake can be observed in both normal and aneurysmal aortic walls, and therefore, not correlated to the aneurysm size. However, different studies showed contradictory findings in terms of correlation between [^18^F]-FDG uptake and AAA expansion or risk of rupture.^14^,^15^ The use of [^18^F]-FDG PET for AAA imaging is therefore limited, with potential confounding factors and lack of specificity, thereby raising concerns about its future clinical use in predicting potential AAA expansion and risk of rupture.^14^,^15^ Nevertheless, an alternative PET radiotracer, [^18^F]-NaF, is currently being explored as a marker for microcalcification in the cardiovascular system^16^–^18^ and has been used to investigate coronary atherosclerosis,^19^,^20^ abdominal atherosclerosis,^21^ aortic stenosis^22^,^23^ and AAA diseases.^24^ Preliminary investigation^24^ shows that this tracer is promising for improved prediction of AAA disease progression, and may therefore facilitate early intervention for those at higher risk of rupture. However, a major confounding issue is the artificial spill in contamination from the bone into the aneurysm due to the limited PET resolution and the associated partial volume effect. [^18^F]-NaF is predominantly taken up by bone,^19^,^20^,^24^ thus the AAA regions in close proximity to the bones have considerably higher uptake than more distal regions.^24^

Common conventional techniques to mitigate the spill in contamination include masking out the high uptake region in the image space, or simply excluding areas of spill in from regions of interest during image analysis.^24^ The obvious challenge in these techniques is the high dependence of the measurements on clinician subjective choices. In addition, a certain degree of potentially important physiological information may be lost from the excluded regions. This is because the posterior retroperitoneal rupture (i.e. rupture from the aneurysm site close to the bone) is the most common and which could be treated with early clinical intervention.^1^,^25^ These issues clearly suggest the need for a more objective method to correct for the spill in effects. Therefore, the aim of this study is to investigate the spill in from the bone into the aneurysm and its effect on AAA quantification and patient management. We also aim to correct for the spill in effects using a recently proposed background correction technique, and then compare its performance against the current approach of simply excluding the part of the aneurysm that is close to the bone. To the best of our knowledge, no previous study has been performed on estimating and correcting for the spill in effect in [^18^F]-NaF imaging of AAA.

## Materials and Methods

### Datasets

Seventy-two (72) PET/computed tomography (PET/CT) datasets from patients with varying aneurysm diameters were used from the archive of the [^18^F]-sodium fluoride uptake in abdominal aortic aneurysm (SoFIA^3^) PET/CT study (NCT02229006).^24^ The study involved patients older than 50 years with asymptomatic AAA (larger than 40 mm anteroposterior diameter) who have been under routine clinical surveillance. The data consists of 61 males and 11 females with age range 72.5 ± 6.9 years, body mass index 27.6 ± 3.5 kg/m^2^ and aortic diameter 48.8 ± 7.7 mm. Each patient was injected with 125 MBq of [^18^F]-NaF and imaged 60 minutes post-injection on the Biograph mCT^™^ scanner (Siemens Healthineers, Knoxville, TN, USA).^26^ A low-dose CT attenuation correction (CTAC) scan was performed (120 kV, 50 mAs, 5/3 mm) followed by acquisition of PET data using 3 × 10 min bed positions to ensure coverage from the thoracic aorta to the aortic bifurcation.

All patients gave their written informed consent, and approval was given by the research ethics committee in accordance with the Declaration of Helsinki.

### Reconstruction and Spill in Correction

The data were reconstructed using the software for tomographic image reconstruction (STIR) library^27^ with the ordered subset expectation maximization (OSEM) algorithm (21 subsets, 3 iterations). Additionally, point spread function (PSF) modelling was incorporated into the reconstruction as an isotropic 3D Gaussian kernel with 4.4 mm full width at half maximum (FWHM) in both axial and transverse planes.^28^ The spill in effect from the bone into the aneurysm was corrected using a previously proposed background correction (BC) technique.^29^–^31^ More information about the technique can be seen in the supplementary material. All resulting reconstructed images were post-filtered with an isotropic 3 mm FWHM Gaussian filter.

### Image Analysis

All reconstructed images (OSEM, OSEM + PSF and OSEM + PSF + BC) were analysed using AMIDE.^32^ Region of interest (ROI) analysis was performed using two ROIs: (i) an ellipsoidal ROI over the entire aneurysm (AAA), and (ii) another ellipsoidal ROI over the aneurysm but excluding the part close to the bone (AAA_exc_). Information about the exclusion criteria for AAA_exc_ can be found in the Supplementary material. All ROIs were drawn on the CTAC images, and then transferred to the PET images. For both ROIs, the maximum standard uptake values (SUV) were recorded for the entire aneurysm. An ROI was also drawn on the normal aorta (non-AAA) to investigate if there is a significant uptake in the aneurysm compared with the normal aorta. It is useful to note that the AAA is normally expected to have a clinically significant uptake when the % uptake difference between AAA and non-AAA is greater than 25%.^19^,^33^,^34^ Following standard clinical quantification methods,^24^,^35^–^37^ we estimated the corrected maximum SUV $({\text{cSUV}}_{\hbox{max} } )$, and target-to-blood ratio $({\text{TBR}}_{\hbox{max} } )$ using:

1 $$ {\text{cSUV}}_{\hbox{max} } = {\text{SUV}}_{\hbox{max} } ({\text{T}}) - {\text{SUV}}_{\text{mean}} ({\text{B}}) $$

2 $$ {\text{TBR}}_{\hbox{max} } = \frac{{{\text{SUV}}_{\hbox{max} } \left( {\text{T}} \right)}}{{{\text{SUV}}_{\text{mean}} \left( {\text{B}} \right)}} $$

where ${\text{SUV}}_{ \hbox{max} } ({\text{T}})$ correspond to the maximum SUV in the target aneurysm region, while ${\text{SUV}}_{\text{mean}} ({\text{B}})$ is the mean SUV in the background (blood pool region). The blood pool SUV was taken as the mean uptake in 2 cm^2^ ellipsoidal ROIs placed on three consecutive slices at the right atrium.

### Statistical Analysis

Statistical analysis was performed using the IBM SPSS statistics software package, version 23. Pearson’s correlation analysis was performed to investigate the correlation between [^18^F]-NaF uptake in the aneurysm and AAA diameter, for OSEM, OSEM + PSF and OSEM + PSF + BC images. The significance of the uptake differences between the uncorrected and corrected images and between the two ROI groups (AAA and AAA_exc_) for all reconstruction methods was compared using a paired *t* test. A *P* value less than .05 was considered statistically significant.

Finally, a direct comparison was made between the conventional quantification technique (i.e. OSEM + PSF (AAA_exc_)) and the background correction technique (OSEM + PSF + BC (AAA)). The relative difference in the uptake values between these techniques was given as:

3 $$ \% \,{\text{difference}} = \frac{{{\text{OSEM}} + {\text{PSF}} + {\text{BC}}({\text{AAA}}) - {\text{OSEM}} + {\text{PSF}}({\text{AAA}}_{\text{exc}} )}}{{{\text{OSEM}} + {\text{PSF}}({\text{AAA}}_{\text{exc}} )}} \times 100 $$

These values are expressed as mean, standard deviation (SD) of the difference, and 95% confidence interval (CI) and a Bland–Altman analysis was carried out on the data. Changes larger than 25% are considered clinically significant based on EORTC specification.^38^ Single-measure intraclass correlation coefficient (ICC) and Cronbach’s *α* statistics were used as measures of absolute agreement and reliability between the two techniques. ICC ranges from 0 to 1, with values closer to 1 representing better reproducibility.^39^

## Results

**Figure 1:**
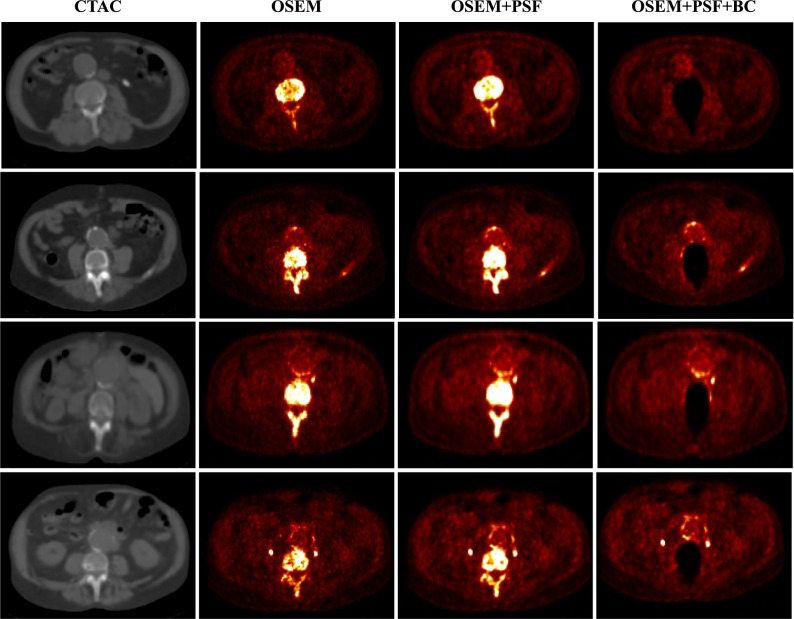
CT images and PET reconstructed images of four indicative patient datasets, showing a high [^18^F]-NaF uptake in the bone and the aneurysm. The activity contribution from the bone was removed in OSEM + PSF + BC

This section presents the quantification results of the aneurysm and normal aorta obtained from all the reconstruction algorithms. Figure 1 shows the images as reconstructed from all three reconstruction algorithms which indicate a high [^18^F]-NaF uptake in the aneurysm and the bone. Note that the bone uptake has been removed in the OSEM + PSF + BC images.

### [^18^F]-NaF Uptake in Aneurysm (AAA) and Normal Aorta (Non-AAA)

**Figure 2:**
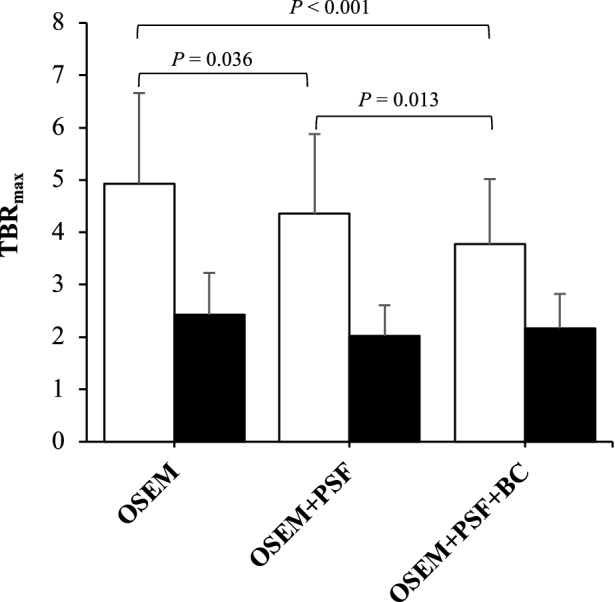
Evaluating the significance of uptake differences between the uncorrected and corrected images using paired *t* test. The plot displays the mean TBR_max_. The error-bar represents the standard deviation (SD)

For all the patient data involved in the study, there is a higher [^18^F]-NaF uptake (quantified as SUV_max_) in the aneurysm (AAA) than in the normal aorta, as shown in Figure 2. For all the algorithms, the mean TBR_max_ is not significantly different for the normal aorta, but it is significantly different for the AAA.

The t test conducted on the reconstruction algorithms showed that there is a significant difference in AAA TBR_max_ between all algorithms. There is also a statistically significant difference in the AAA TBR_max_ between OSEM + PSF and OSEM + PSF + BC. However, for the normal aorta, there is no significant difference between the uptake values of the different reconstruction methods.

### Correlation Between [^18^F]-NaF Uptake and AAA Diameter

Table 1 shows the results of the correlation analysis performed on images reconstructed with OSEM, OSEM + PSF and OSEM + PSF + BC to investigate the correlation between [^18^F]-NaF uptake and AAA diameter. For all the reconstruction algorithms, no significant correlation was observed.

**Table 1:** Analysis of correlation between [^18^F]-NaF uptake and AAA diameter

	SUV_max_	cSUV_max_	TBR_max_
OSEM			
Pearson’s *R*	0.13	0.11	0.05
*P* value	.22	.36	.44
OSEM + PSF			
Pearson’s *R*	0.10	0.11	0.07
*P* value	.11	.37	.16
OSEM + PSF + BC			
Pearson’s *R*	0.08	0.08	0.05
*P* value	.26	.53	.64

### AAA Uptake Differences Due to ROI Selection

Table 2 shows the SUV_max_, cSUV_max_ and TBR_max_ of the AAA and AAA_exc_ calculated using Eqs. 1 and 2. It can be seen that there is an uptake difference between AAA and AAA_exc_ for all the reconstruction algorithms. With AAA_exc_, OSEM + PSF and OSEM + PSF + BC have the same SUV_max_ (2.73) and closely related cSUV_max_ and TBR_max_, but with the whole AAA, all algorithms produce significantly different values, with OSEM giving the highest TBR_max_ (4.93 ± 1.73), while OSEM + PSF + BC the lowest (3.77 ± 1.25). For SUV_max_, cSUV_max_ and TBR_max_, the difference in quantification between AAA and AAA_exc_ is statistically significant for OSEM and OSEM + PSF, but not for OSEM + PSF + BC. Also, OSEM + PSF + BC showed the least difference between AAA and AAA_exc_, and it also had the least SD across all the quantification metrics used.

**Table 2:** The SUV_max_, cSUV_max_ and TBR_max_ of the aneurysm (AAA) for all the reconstruction algorithms

	Mean ± SD		*P* value
	AAA	AAA_exc_	
SUV_max_			
OSEM	3.32 ± 1.05	2.75 ± 0.84	.00038
OSEM + PSF	3.62 ± 1.30	2.73 ± 0.79	< .0001
OSEM + PSF + BC	2.85 ± 0.89	2.73 ± 0.81	.40
cSUV_max_			
OSEM	2.61 ± 0.97	2.03 ± 0.75	.0001
OSEM + PSF	2.75 ± 1.19	1.86 ± 0.68	< .0001
OSEM + PSF + BC	2.05 ± 0.77	1.93 ± 0.70	.34
TBR_max_			
OSEM	4.93 ± 1.73	4.08 ± 1.44	.0018
OSEM + PSF	4.36 ± 1.52	3.30 ± 1.02	< 0.0001
OSEM + PSF + BC	3.77 ± 1.25	3.63 ± 1.22	0.48

The SUVs of AAA were extracted using two ROIs (AAA and AAA_exc_) in order to quantify the spill in effect from the bone. The differences between AAA and AAA_exc_ were compared using a paired t test. Values are expressed as mean ± standard deviation (SD). A *P* value less than .05 was considered statistically significant

It could also be seen (in Table 3) that while using AAA_exc_, the mean % uptake difference ($\bar{d}$) between the aneurysm and normal aorta is about 70% for all algorithms, the same difference, using AAA is much higher for OSEM and OSEM + PSF images than OSEM + PSF + BC (OSEM ≈ 110%, OSEM + PSF ≈ 123% and OSEM + PSF + BC ≈ 79%). The 95% limit of agreement of $|\bar{d}|$, defined as ${\text{LOA}} = \bar{d} \pm 1.96{\text{SD}}$ is also higher in OSEM and OSEM + PSF images than OSEM + PSF + BC images. There is also a major difference in the number of patients appearing to exhibit a significant AAA uptake (as depicted by a % difference higher than 25%). With AAA_exc_, about 90% (85% and 86% for OSEM + PSF and OSEM + PSF + BC reconstructions, respectively) of the patients have significant uptake in the aneurysm, whereas with AAA, we have 97% in OSEM and OSEM + PSF, and 90% in OSEM + PSF + BC.

**Table 3:** The analysis of the % uptake difference (*d*) between aneurysm (AAA or AAA_exc_) and normal aorta (non-AAA) using the TBR_max_. $\bar{d}$ is the mean % uptake difference for all the patients, and LOA is the 95% Limit of Agreement of $|\bar{d}|$, defined as ${\text{LOA}} = \bar{d} \pm 1.96{\text{SD}}$

	Mean % difference, $\bar{d}$	SD	LOA	No of patients with *d* > 25% (%)
OSEM				
AAA	110.5	65.8	− 18.5 to +239.6	70 (97)
AAA_exc_	72.7	44.9	− 15.1 to +160.7	65 (90)
OSEM + PSF				
AAA	123.1	76.1	− 26.1 to +272.3	70 (97)
AAA_exc_	67.8	43.4	− 17.3 to +152.8	61 (85)
OSEM + PSF + BC				
AAA	79.1	47.2	− 13.5 to +171.6	65 (90)
AAA_exc_	72.2	45.3	− 16.5 to +160.9	62 (86)

**Figure 3:**
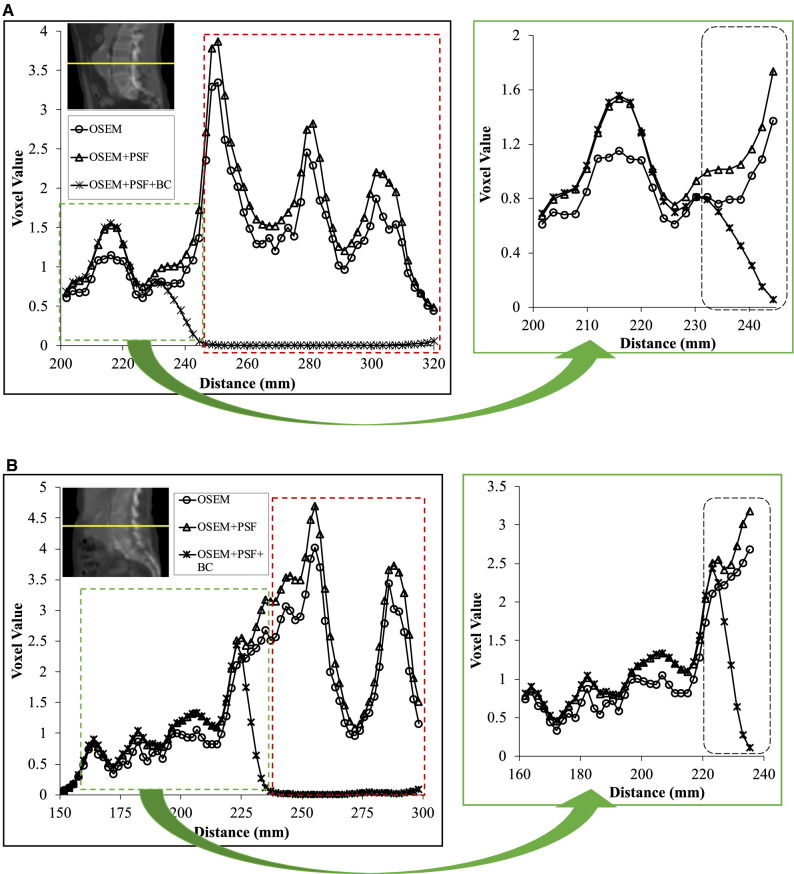
Profile across the bone (red dashed rectangle) and the aneurysm (green dashed rectangle), when the aneurysm is (**A**) detached from the bone, and (**B**) in contact with the bone. The portion of the aneurysm prone to the spill in effects from the bone is highlighted by the black dashed rectangle. Note that for OSEM + PSF + BC, the bone activity has been removed

The disparity in quantification between AAA and AAA_exc_ is partly due to the spill in effect from the bone into the aneurysm, as shown with line profiles shown for the reconstructed images in Figure 3. When the aneurysm is detached from the bone (Figure 3A), the maximum voxel value is 1.73 in the spill in prone area, and 1.56 in the rest of the aneurysm. This implies that the spill in effect can potentially increase the SUV_max_ in the aneurysm by a factor of 1.09. However, when the aneurysm is in contact with the bone (Figure 3B), the maximum voxel value is 3.18 in the spill in prone area, and 2.09 in the rest of the aneurysm resulting in a spill in factor of about 1.52. This spill in effect varies in magnitude with the relative position of the aneurysm to the bone as aneurysms in close distance to the bone suffer more spill in effect than farther aneurysms.

### Comparison Between OSEM + PSF (AAA_exc_) and OSEM + PSF + BC (AAA)

**Figure 4:**
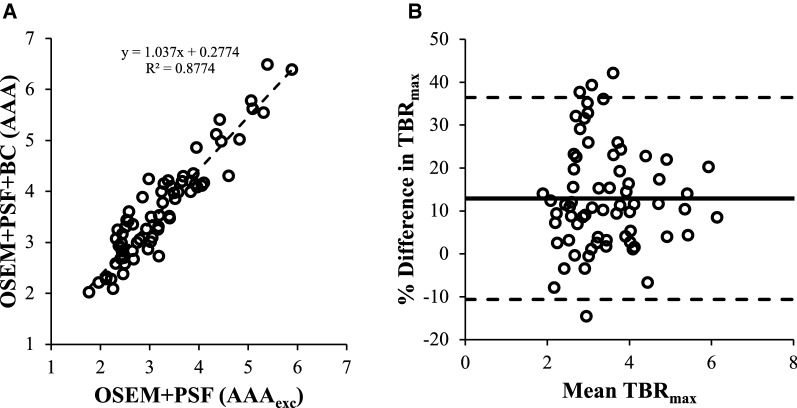
Correlation analysis between the conventional quantification approach (OSEM + PSF (AAA_exc_)) and the background correction approach (OSEM + PSF + BC (AAA)): (**A**) shows a good correlation between the two techniques, and (**B**) is a Bland–Altman plot showing the level of agreement between the two techniques. The continuous line shows the % bias while the dashed lines represent the upper and lower LOA

The percentage difference in OSEM + PSF + BC (AAA) values with respect to OSEM + PSF (AAA_exc_) was estimated using a Bland–Altman plot as displayed in Figure 4. The mean difference, SD and 95% CI and the correlation between the two techniques (using single-measure ICC and the Cronbach’s *α* statistics) are presented in Table 4.

**Table 4:** Correlation and repeatability test between OSEM + PSF (AAA_exc_) and OSEM + PSF + BC (AAA)

	SUV_max_	cSUV_max_	TBR_max_
Mean difference (%)	3.37	9.49	12.90
SD	7.26	12.44	12.01
No. with *d* > 25% (%)	3 (4)	7 (10)	11 (15)
Intraclass correlation	0.93	0.88	0.83
95% CI	0.87 to 0.96	0.71 to 0.94	0.36 to 0.93
Cronbach’s *α*	0.97	0.95	0.95

Most of the points lie within the 25% difference, except for few ones as reported in Table 4. These results show a good conformity with the EORTC specification. The results also show excellent correlations between the two reconstruction methods. There is also a high reliability and reproducibility between the two methods.

There is, however, a difference in quantification between the two methods, as the uptake positivity in aneurysm change from insignificant (with OSEM + PSF) to positively significant (with OSEM + PSF + BC) in four patients, and from positive to negative in 1 patient. Details are shown in Supplementary Table S1.

## Discussion

[^18^F]-NaF PET imaging is currently being explored as a promising imaging biomarker for microcalcification in abdominal aortic aneurysms (AAA). However, a confounding issue is the spill in contamination from the bone into the aneurysm. Therefore, this study has investigated the spill in effect in [^18^F]-NaF PET imaging of the abdominal aortic aneurysms and its dependence on the AAA ROI delineation method. We also evaluated the performance of the background correction technique aimed at reducing the spill in effect regardless of the AAA ROI delineation method.

For all the patient data involved in the study, there was a significant [^18^F]-NaF uptake in the aneurysms. However, the Pearson correlation analysis performed on all the reconstructed images showed that there was no correlation between [^18^F]-NaF uptake and AAA diameter for any of the algorithms (Table 1) as reflected in the SoFIA^3^ study.^24^ The study however showed an indication that [^18^F]-NaF may have the ability to stratify high-risk aneurysms even before rupture. Therefore, better AAA disease prediction using [^18^F]-NaF, in addition to clinical risk factors including AAA diameters, would be of great benefit to patients with high-risk aneurysms which size may be smaller than what the current guidelines may suggest (i.e. 55 mm).

Furthermore, all reconstruction algorithms demonstrated a higher [^18^F]-NaF uptake in the aneurysms than in the healthy part of the aorta, as illustrated in Figure 2. The mean TBR_max_ for the normal aorta is almost the same for all the images, whereas for the aneurysm, the TBR_max_ is different for all images, with the OSEM algorithm producing images with the highest TBR_max_. The paired t test showed that there was a significant difference in AAA TBR_max_ for all reconstructed images. There is also a statistically significant difference in the AAA TBR_max_ between OSEM + PSF and OSEM + PSF + BC. We also found a statistically significant uptake difference in the aneurysm between AAA and AAA_exc_. OSEM + PSF and OSEM + PSF + BC exhibited almost the same TBR_max_ in AAA_exc_, but OSEM yielded the highest TBR_max_ in AAA, while OSEM + PSF + BC attained the lowest uptake. While quantifying with SUV_max_, PSF-based reconstructions produced the highest value, and this could be attributed to the commonly reported Gibbs artefacts, resulting in an overshoot around the hot region (i.e. bone).^40^,^41^ This also led to a considerably higher difference in uptake between AAA and AAA_exc_ ROIs, relative to OSEM, thereby suggesting, a higher spill in effect with OSEM + PSF than OSEM reconstruction. However, the spill in correction effectively attained by the added application of the background correction technique eliminated the overestimation and ROI-induced variability effect due to PSF modelling, thereby yielding similar SUV_max_ and TBR_max_ scores regardless of the ROI delineation method (AAA or AA_exc_). For SUV_max_, cSUV_max_ and TBR_max_, the difference in quantification between AAA and AAA_exc_ was statistically significant for OSEM and OSEM + PSF, but not for OSEM + PSF + BC (Table 2). In addition, OSEM + PSF + BC exhibited the least mean and SD differences between AAA and AAA_exc_ across all the quantification metrics used.

Although the use of AAA_exc_ ROIs revealed % difference between aneurysm and normal aorta that were within 70% for all algorithms, a large disparity was found when quantifying with the AAA ROIs. This naturally led to major differences between the BC and non-BC methods in the number of patients having a significant AAA uptake, as shown in Table 3. With AAA_exc_, about 90% (85%-86% in both PSF-based reconstructions) of the patients exhibited significant uptake in the aneurysm, whereas with AAA, the respective % of patients were 97% in OSEM and OSEM + PSF but only 90% in OSEM + PSF + BC. So with AAA and AAA_exc_, the net difference in the number of patients with significant PET uptake is 7%, 12% and 4% in OSEM, OSEM + PSF and OSEM + PSF + BC, respectively. This significant disparity between the two ROIs was partly due to the spill in effect emanating from the adjacent bone into the aneurysm, as demonstrated in Figure 3. This spill in effect varied in magnitude with the position of the aneurysm relative to the bone, as aneurysms is close to the bone are expected to be more susceptible to the spill in artefacts from the bone. Thus, a reasonable strategy to mitigate these artefacts would be to exclude the parts of the AAA region located close to the bone during image analysis. However, the obvious risk of such an approach would then be the elimination of a certain degree of potentially important physiological information due to the exclusion of these AAA regions. In particular, the posterior retroperitoneal rupture which is the most common type of AAA rupture and could be treated with early clinical intervention, is usually located at the aneurysm site close to the bone.^1^,^25^ Furthermore, in the patient cases presented in Figure 1, regions in posterior parts of AAA close to the bone were identifiable with their genuine CT and PET signal in the posterior parts of the aneurysm which would be independent from the spill in signal from the neighbouring bone.

Comparison of the conventional quantification approach (OSEM + PSF (AAA_exc_)) with the background correction approach (OSEM + PSF + BC (AAA)) shows that there is an excellent correlation between the two methods (Figure 4 and Table 4). The Bland–Altman analysis shows the % mean difference of 3%, 9% and 13% for SUV_max_, cSUV_max_ and TBR_max_, respectively. This difference is due to the differences in ROI delineation as some signals may have been removed from the aneurysm using the AAA_exc_ approach, which leads to a significant change in four patients as TBR_max_ changes from insignificant uptake (using OSEM + PSF (AAA_exc_)) to positively significant (using OSEM + PSF + BC (AAA)). This result is available in the Supplementary Table 1. However, it could be seen that most of the values displayed on the Bland–Altman plots still lie within the 25% difference which shows a good conformity with the EORTC specification.^38^ There is also high reliability and reproducibility between the two methods. In essence, OSEM + PSF + BC (AAA) can be used in place of OSEM + PSF (AAA_exc_). In this way, the risk of removing indicative physiological uptake from the aneurysm due to ROI selection will be eliminated. Moreover, an automated aneurysm ROI can be drawn on the OSEM + PSF + BC image, without much effort to manually exclude the aneurysm part close to the bone.

Although the BC technique was used in this study to effectively remove the spill in activity from the bone into the aneurysm, the application is not limited to aneurysm imaging, but in principle it can be applied to other mappable regions such as the aortic valves^19^ and the mitral annulus^42^ where an automated (or semi-automated) ROI can be drawn on the BC image without much effort to manually exclude the uptake from the hot region. Further study is, however, needed to validate this. In addition, this study was done with [^18^F]-NaF PET/CT where the bone was segmented from the CTAC image. The clinical translation of the BC technique might be challenging for [^18^F]-NaF PET/MR imaging in terms of the anatomical segmentation of the bone. An alternative approach will then be to segment the bone from the PET image^43^ but this will require a more careful implementation as the segmented bone might also include the spill in-prone regions of the aneurysm.

## Study Limitations and Future Work

A limitation of this study is that there was no time-of-flight (TOF) implementation, even though the mCT scanner supports TOF. Although TOF has been shown to mitigate errors due to data inconsistency,^44^ there is no clear indication that TOF implementation can sufficiently correct for the spill in effect, especially for regions close to active regions such as the bone. In fact, our past study^31^ has shown that TOF could not sufficiently correct for the spill in effects in lesions adjacent to a hot background region. We could also see that the PSF implementation in this study was unable to fully correct for the spill in effect emanating from the bone into the aneurysms (Tables 1 and 2). This could partly be due to the fact that a spatially invariant PSF approximation was used in this study, and this may not work properly when quantifying regions far from the center of the transaxial field of view.^40^ This limitation could be addressed with accurate modelling of the PSF.^45^ In future, it will be useful to include follow-up datasets and carry out an inter-observer variability study to assess the effect of AAA_exc_ ROI delineation method on the reproducibility of OSEM + PSF TBR_max_ assessments against the effect of CTAC-based bone segmentation method on the reproducibility of OSEM + PSF + BC TBR_max_ assessments. It will also be interesting to carry out radiomic analysis study where the proposed method is expected to offer a larger uptake area to evaluate its characteristics.

## New Knowledge Gained

In this study, we have shown that the spill in effects from the bone leads to overestimation of quantitative values in the aneurysm. This varies with the relative distance between the aneurysm and the bone, as aneurysms close to the bone may have their SUV_max_ overestimated up to a factor of 1.5. We have also shown that the spill in effect is further influenced by the differences in the ROI selection criteria. The two ROIs (AAA and AAA_exc_) used in this study resulted in a net difference in the number of patients with significant PET uptake of 7%, 12% and 4% with OSEM, OSEM + PSF and OSEM + PSF + BC, respectively. However, the background correction (BC) technique is more robust to differences in the ROI delineation criteria and is effective in correcting for the spill in effect from the bone, thereby enhancing accurate quantification at the aneurysm. There is also a possible indication that the BC technique might help in improving patient management and treatment decision if successfully incorporated into clinical routine as demonstrated by the four patients cases where the TBR_max_ originally showed an insignificant uptake with the OSEM + PSF (AAA_exc_) method, but changed to positively significant when using OSEM + PSF + BC (AAA) technique.

## Conclusion

We have evaluated the performance of the background correction (BC) technique in improving quantification and correcting for the spill in effect in [^18^F]-NaF PET/CT imaging of the abdominal aortic aneurysm (AAA). This study showed that the BC technique is less susceptible to differences in ROI delineation criteria and could, therefore, effectively correct for the spill in effect from the bone into the aneurysm.

## Electronic supplementary material

Below is the link to the electronic supplementary material.

Supplementary material 1 (DOCX 348 kb)

Supplementary material 2 (PPTX 1994 kb)

Supplementary material 3 (MP3 5368 kb)

## Abbreviations

AAA: Abdominal aortic aneurysm
PET: Positron emission tomography
CT: Computed tomography
BC: Background correction
SUV: Standardised uptake values
[18F]-NaF: [18F]-sodium fluoride
ROI: Region of interest
STIR: Software for tomographic image reconstruction
FWHM: Full width at half maximum
PSF: Point-spread function
OSEM: Ordered subset expectation maximisation
