# Supplementary material for: Iterative reconstruction incorporating background correction improves quantification of [18F]-NaF PET/CT images of patients with abdominal aortic aneurysm
Source: J Nucl Cardiol. 2019 Nov 11;28(5):1875–86. doi: 10.1007/s12350-019-01940-4 (PMC8648624; doi:10.1007/s12350-019-01940-4)
Supplement: Supplementary file 1 — Supplementary material 1 (DOCX 348 kb) [file 12350_2019_1940_MOESM1_ESM.docx]

**Iterative reconstruction incorporating background correction improves quantification of [^18^F]-NaF PET images of patients with abdominal aortic aneurysm**

Mercy I. Akerele, MSc^a^, Nicolas A. Karakatsanis, PhD^b^, Racheal O. Forsythe, MD^c,d^; Marc R. Dweck, MD, PhD^c,d^, Maaz Syed, MD, PhD^c,d^, Robert G. Aykroyd, PhD^e^, Steven Sourbron, PhD^a^, David E. Newby, MD, PhD^d^, Charalampos Tsoumpas, PhD^a^

^a^ Biomedical Imaging Science Department, Leeds Institute of Cardiovascular and Metabolic Medicine, University of Leeds, Leeds, UK.

^b^ Division of Radiopharmaceutical Sciences, Department of Radiology, Weil Cornell Medical College of Cornell University, NY, USA.

^c^ British Heart Foundation Centre for Cardiovascular Science, University of Edinburgh, Edinburgh, UK.

^d^ Edinburgh Imaging Facility, Queen’s Medical Research Institute, University of Edinburgh, Edinburgh, UK.

^e^ Department of Statistics, University of Leeds, Leeds, UK.

1. ***Background Correction Method***

This method involves segmentation of the background region (bone) from a high-resolution anatomical image such as MR or CT. The region mask $S_{j}$is multiplied by the reconstructed image $f_{j}^{\left( n \right)}$ at three iterations to obtain the background contribution $B_{j}=S_{j}f_{j}^{\left( n \right)}$ in the reconstructed image. This is forward-projected to obtain the sinogram $\left( P_{i}=\sum_{j} H_{ij}B_{j} \right)$, which is then added to the original additive term $A_{i}$ of the OSEM+PSF reconstruction to obtain a corrected image. The flow chart of the algorithm is illustrated in Fig. 1 and defined by Eq. (1):

$f_{j}^{\left( n+1 \right)}= \frac{f_{j}^{\left( n \right)}}{\sum_{i\in J_{j}} H_{ij}}\sum_{i\in J_{j}} H_{ij}\frac{y_{i}}{\sum_{k\in I_{i}} H_{ik}f_{k}^{\left( n \right)}+ A_{i}+ P_{i}}$ (1)

where $y_{i}$is the recorded projection data for detector pair *i*, $H_{ij}$ is the element of the system matrix defining the probability of an event located in image pixel *j* being detected by detector pair *i*, $M_{i}$ and $A_{i}$ are the multiplicative and additive sinogram terms, respectively, $P_{i}$ is the background sinogram contribution, added to the original additive term $A_{i}$ of the standard OSEM+PSF reconstruction, and $f_{j}^{\left( n+1 \right)}$ is the background-corrected activity at voxel *j* of the reconstructed image updated after the *n^th^* iteration.

**Fig. S1.** The flowchart of the background correction (BC) technique.

1. ***ROIs for the Spill-in Estimation***

The SUVs at the aneurysm were estimated by delineating two ROIs: ROI_1_ was delineated over the entire aneurysm region (AAA), while ROI_2_ was delineated such that it excludes regions proximal to the active bone (AAA_exc_). According to past research (1) which showed that the spill in effect is pronounced within two voxels to the active region (in this case, bone), the AAA_exc_ was drawn such that its distance from the bone is 5mm (corresponding to about 2 voxels). This is demonstrated in Figure S2. These ROIs were drawn on the CTAC image (as shown in Fig. S2) and then applied to the PET images.

**Fig. S2:** The transverse and sagittal views of the CTAC image, showing the ROIs used to extract the SUVs at the aneurysm. The outer and inner ROIs represent AAA and AAA_exc_, respectively. AAA_exc_ is drawn such that its distance from the bone is approximately 4mm.

1. ***Correlation between*** ***[^18^F]-NaF uptake and AAA diameter***

Figure S3 shows the results of the correlation between [^18^F]-NaF uptake and AAA diameter. Two outliers were excluded from the analysis (as shown by the blue spheres) because they result in a false linear correlation.**Fig. S3** Results of the regression analysis to investigate the relationship between [^18^F]-NaF uptake and AAA diameter. [^18^F]-NaF uptake was quantified in OSEM+PSF+BC images using TBR_max_ with an ROI covering the entire aneurysm (AAA). In the equations, T and D are the AAA TBR_max_ and diameters, respectively.

1. ***Comparison between OSEM+PSF (AAA_exc_) and OSEM+PSF+BC (AAA)***

While the performance of the OSEM+PSF+BC (AAA) and OSEM+PSF (AAA_exc_) seem to be similar, there is a difference in quantification and positivity definition for the aneurysm. This result is presented in Table S1.

**Table S1:** Comparison between OSEM+PSF (AAA_exc_) and OSEM+PSF+BC (AAA) using the TBR_max_. The results highlighted in red show cases where the result has changed from negative (with OSEM+PSF) to positive (with OSEM+PSF+BC) for the corresponding patient while the results in green highlight the cases for which the results have changed from negative (with OSEM+PSF) to positive (with OSEM+PSF+BC).

|  | OSEM+**PSF** | | | OSEM+**PSF+BC** | | |
| --- | --- | --- | --- | --- | --- | --- |
| **No** | **non-AAA** | **AAA_exc_** | **% Difference** | **non-AAA** | **AAA** | **% Difference** |
| **1** | 2.43 | 3.49 | 43.74 | 2.59 | 3.96 | 52.76 |
| **2** | 1.77 | 2.80 | 58.23 | 1.88 | 3.05 | 62.73 |
| **3** | 2.37 | 3.41 | 44.09 | 2.33 | 3.47 | 48.57 |
| **4** | 2.01 | 2.26 | 12.84 | 1.63 | 2.08 | 27.45 |
| **5** | 2.97 | 5.89 | 98.24 | 3.28 | 6.38 | 94.79 |
| **6** | 1.70 | 3.19 | 87.52 | 1.45 | 2.72 | 88.27 |
| **7** | 2.36 | 3.06 | 29.54 | 2.34 | 3.09 | 32.08 |
| **8** | 2.84 | 4.43 | 56.00 | 3.49 | 5.40 | 54.48 |
| **9** | 2.48 | 3.03 | 22.08 | 2.94 | 3.49 | 18.81 |
| **10** | 1.86 | 3.69 | 97.95 | 2.00 | 4.29 | 114.32 |
| **11** | 2.08 | 2.57 | 23.59 | 2.44 | 3.41 | 39.88 |
| **12** | 3.21 | 4.82 | 50.06 | 3.19 | 5.01 | 56.91 |
| **13** | 1.53 | 2.44 | 59.84 | 1.64 | 2.82 | 72.02 |
| **14** | 1.94 | 2.58 | 33.33 | 2.11 | 3.60 | 70.45 |
| **15** | 2.50 | 3.95 | 58.46 | 3.16 | 4.85 | 53.38 |
| **16** | 2.16 | 3.83 | 77.63 | 1.90 | 3.99 | 109.19 |
| **17** | 2.06 | 3.89 | 89.06 | 2.22 | 4.34 | 95.53 |
| **18** | 2.20 | 3.97 | 80.75 | 2.25 | 4.07 | 81.36 |
| **19** | 2.26 | 3.18 | 40.63 | 2.44 | 3.30 | 35.19 |
| **20** | 2.79 | 5.39 | 93.31 | 3.46 | 6.48 | 87.52 |
| **21** | 2.24 | 3.02 | 35.11 | 2.25 | 3.01 | 33.74 |
| **22** | 1.50 | 4.36 | 191.12 | 1.68 | 5.11 | 204.32 |
| **23** | 1.54 | 1.77 | 14.65 | 1.75 | 2.02 | 15.19 |
| **24** | 1.59 | 3.24 | 103.26 | 2.04 | 3.99 | 95.29 |
| **25** | 2.81 | 5.32 | 89.36 | 2.88 | 5.54 | 92.59 |
| **26** | 3.97 | 4.46 | 12.43 | 3.99 | 4.97 | 24.68 |
| **27** | 1.60 | 2.12 | 32.47 | 1.86 | 2.32 | 24.82 |
| **28** | 2.33 | 3.20 | 37.30 | 2.44 | 3.52 | 44.25 |
| **29** | 3.40 | 4.61 | 35.58 | 2.98 | 4.29 | 44.02 |
| **30** | 1.55 | 2.75 | 77.10 | 1.57 | 2.99 | 89.59 |
| **31** | 1.19 | 2.12 | 78.86 | 1.24 | 2.27 | 83.09 |
| **32** | 1.17 | 2.41 | 106.96 | 1.17 | 2.89 | 146.55 |
| **33** | 1.33 | 1.97 | 47.85 | 1.44 | 2.21 | 52.95 |
| **34** | 1.38 | 2.32 | 68.75 | 1.60 | 3.07 | 91.81 |
| **35** | 2.02 | 2.31 | 14.46 | 2.28 | 2.58 | 13.08 |
| **36** | 3.26 | 7.25 | 122.58 | 3.83 | 9.15 | 138.56 |
| **37** | 2.02 | 3.58 | 77.11 | 2.28 | 3.98 | 74.61 |
| **38** | 1.13 | 2.65 | 133.71 | 1.06 | 2.83 | 168.11 |
| **39** | 1.49 | 2.98 | 100.80 | 2.12 | 4.24 | 99.73 |
| **40** | 2.49 | 4.10 | 64.49 | 2.85 | 4.16 | 46.12 |
| **41** | 2.15 | 2.45 | 13.85 | 2.37 | 2.74 | 15.72 |
| **42** | 2.18 | 4.07 | 86.97 | 2.25 | 4.11 | 82.91 |
| **43** | 1.36 | 2.22 | 63.22 | 1.31 | 2.28 | 73.41 |
| **44** | 2.28 | 2.58 | 12.97 | 3.19 | 4.58 | 43.73 |
| **45** | 1.62 | 2.38 | 46.95 | 1.77 | 2.93 | 65.81 |
| **46** | 1.33 | 2.45 | 84.42 | 1.50 | 3.16 | 110.32 |
| **47** | 2.04 | 3.40 | 66.79 | 2.12 | 3.50 | 65.51 |
| **48** | 2.31 | 5.06 | 118.83 | 2.53 | 5.77 | 128.36 |
| **49** | 1.44 | 3.17 | 120.96 | 1.46 | 3.25 | 122.35 |
| **50** | 2.22 | 3.67 | 64.77 | 2.39 | 4.19 | 75.24 |
| **51** | 1.67 | 2.94 | 75.47 | 1.81 | 3.25 | 79.71 |
| **52** | 2.09 | 3.27 | 56.55 | 2.09 | 3.77 | 80.73 |
| **53** | 1.90 | 2.97 | 56.17 | 1.81 | 2.86 | 58.21 |
| **54** | 2.84 | 5.09 | 79.15 | 2.95 | 5.62 | 90.67 |
| **55** | 1.13 | 3.83 | 237.85 | 1.23 | 4.20 | 241.34 |
| **56** | 1.57 | 3.44 | 119.51 | 1.93 | 4.09 | 112.55 |
| **57** | 2.83 | 2.35 | -16.84 | 2.92 | 3.24 | 11.00 |
| **58** | 1.60 | 3.29 | 105.40 | 1.74 | 4.14 | 137.52 |
| **59** | 1.91 | 2.46 | 28.94 | 1.78 | 2.38 | 33.24 |
| **60** | 1.81 | 2.48 | 37.03 | 1.86 | 2.69 | 44.90 |
| **61** | 1.94 | 3.93 | 102.17 | 2.07 | 4.14 | 100.27 |
| **62** | 1.50 | 2.43 | 61.76 | 1.51 | 2.98 | 97.23 |
| **63** | 1.28 | 2.68 | 108.99 | 1.27 | 2.66 | 110.48 |
| **64** | 1.89 | 2.66 | 40.72 | 1.79 | 3.35 | 87.22 |
| **65** | 1.90 | 2.51 | 32.04 | 2.59 | 3.30 | 27.26 |
| **66** | 2.54 | 2.86 | 12.59 | 2.80 | 3.88 | 38.92 |
| **67** | 1.56 | 2.49 | 59.42 | 1.54 | 2.57 | 67.15 |
| **68** | 2.07 | 3.52 | 70.54 | 2.23 | 3.85 | 72.80 |
| **69** | 1.92 | 4.19 | 118.16 | 2.08 | 6.84 | 228.83 |
| **70** | 1.34 | 3.38 | 152.22 | 1.62 | 4.20 | 159.21 |
| **71** | 1.56 | 2.42 | 55.26 | 1.72 | 2.68 | 55.39 |
| **72** | 2.38 | 2.54 | 6.55 | 1.75 | 3.43 | 95.66 |
