# Supplementary material for: Iterative reconstruction incorporating background correction improves quantification of [18F]-NaF PET/CT images of patients with abdominal aortic aneurysm
Source: J Nucl Cardiol. 2019 Nov 11;28(5):1875–86. doi: 10.1007/s12350-019-01940-4 (PMC8648624; doi:10.1007/s12350-019-01940-4)
Supplement: Supplementary file 2 — Supplementary material 2 (PPTX 1994 kb) [file 12350_2019_1940_MOESM2_ESM.pptx]

## Slide 1
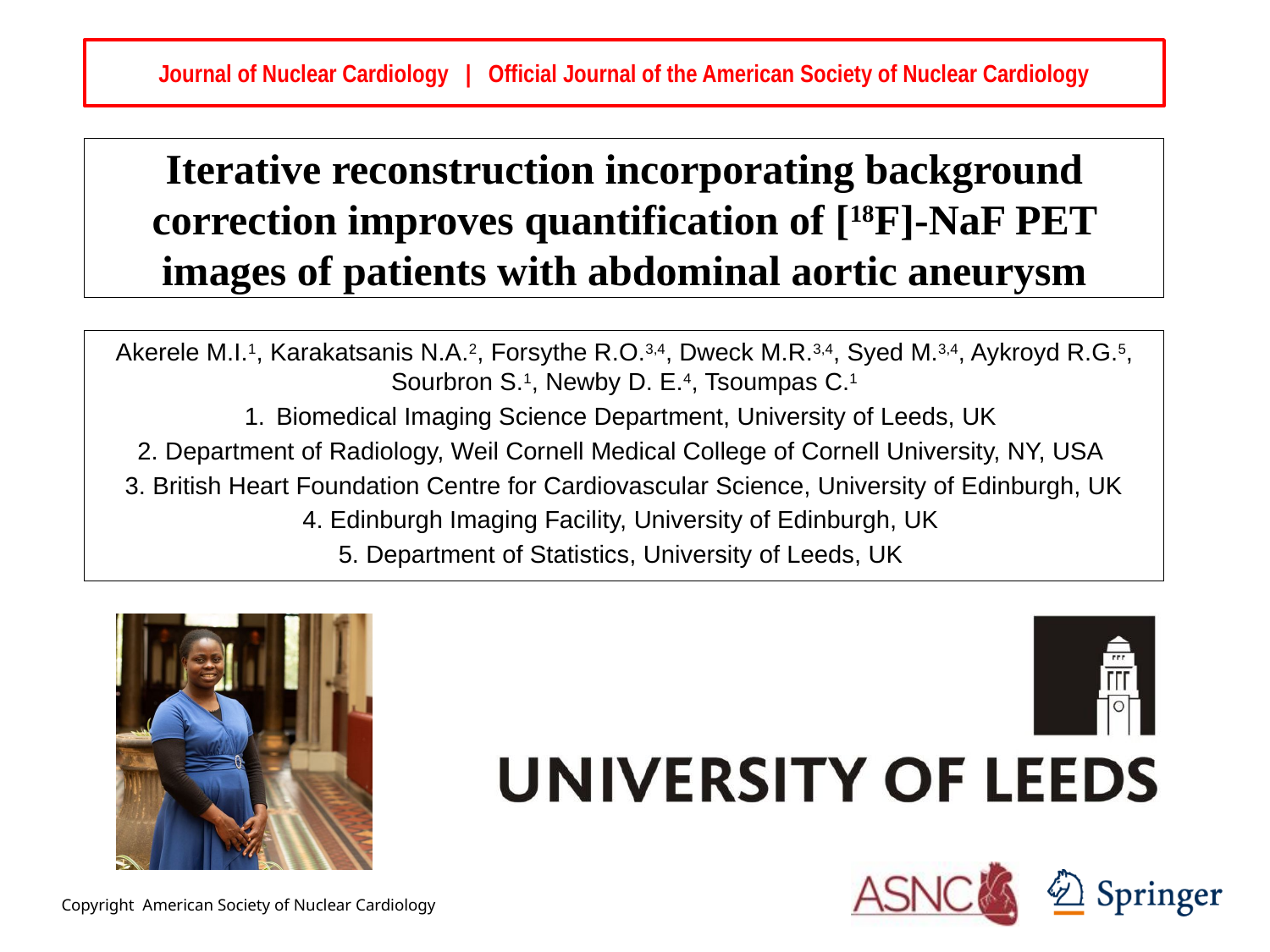

Journal of Nuclear Cardiology | Official Journal of the American Society of Nuclear Cardiology
# Iterative reconstruction incorporating background correction improves quantification of [18F]-NaF PET images of patients with abdominal aortic aneurysm
Akerele M.I.1, Karakatsanis N.A.2, Forsythe R.O.3,4, Dweck M.R.3,4, Syed M.3,4, Aykroyd R.G.5, Sourbron S.1, Newby D. E.4, Tsoumpas C.1
Biomedical Imaging Science Department, University of Leeds, UK
2. Department of Radiology, Weil Cornell Medical College of Cornell University, NY, USA
3. British Heart Foundation Centre for Cardiovascular Science, University of Edinburgh, UK
4. Edinburgh Imaging Facility, University of Edinburgh, UK
5. Department of Statistics, University of Leeds, UK
Copyright American Society of Nuclear Cardiology

## Slide 2
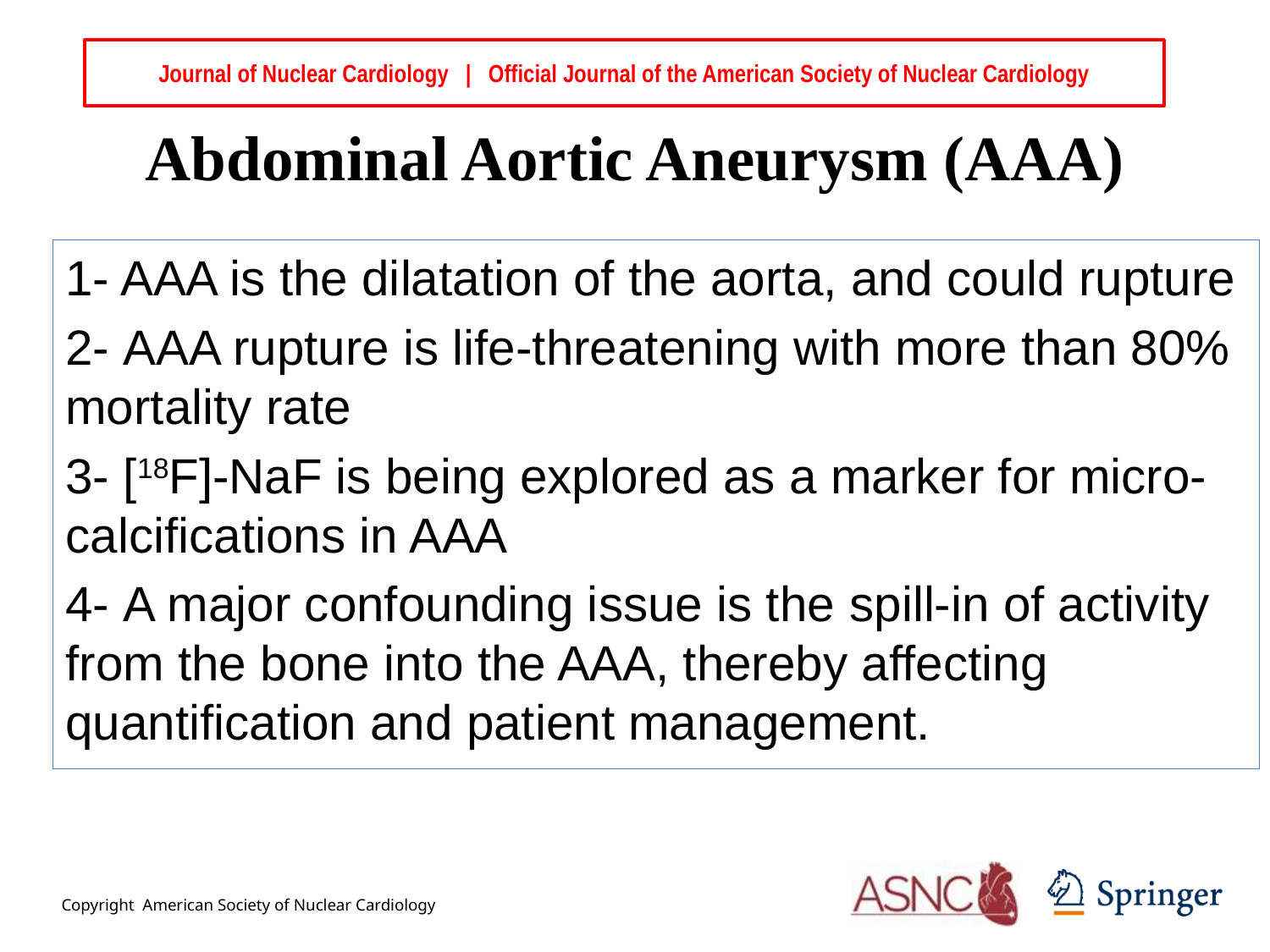

Journal of Nuclear Cardiology | Official Journal of the American Society of Nuclear Cardiology
# Abdominal Aortic Aneurysm (AAA)
1- AAA is the dilatation of the aorta, and could rupture
2- AAA rupture is life-threatening with more than 80% mortality rate
3- [18F]-NaF is being explored as a marker for micro-calcifications in AAA
4- A major confounding issue is the spill-in of activity from the bone into the AAA, thereby affecting quantification and patient management.
Copyright American Society of Nuclear Cardiology

## Slide 3
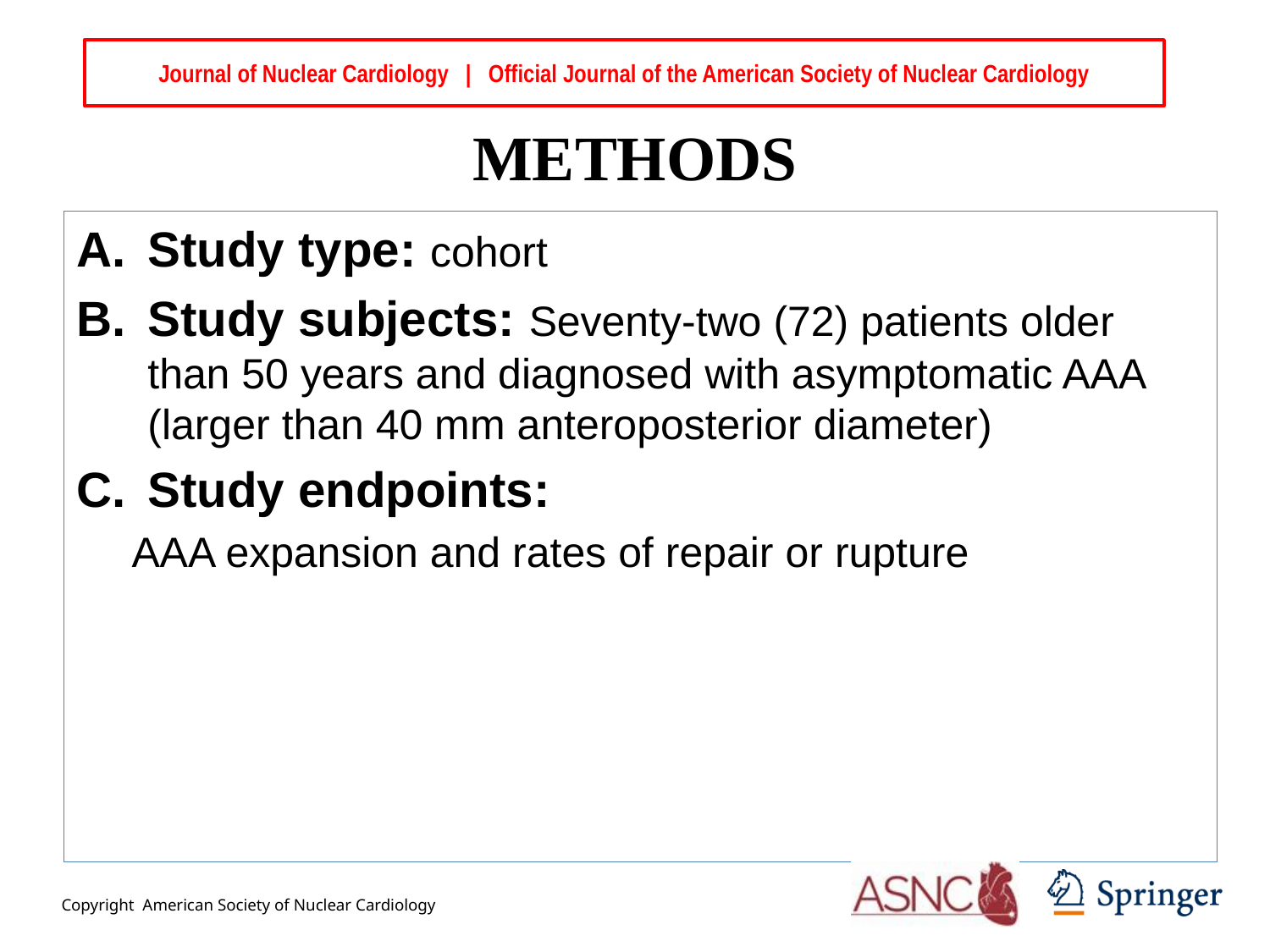

Journal of Nuclear Cardiology | Official Journal of the American Society of Nuclear Cardiology
# METHODS
Study type: cohort
Study subjects: Seventy-two (72) patients older than 50 years and diagnosed with asymptomatic AAA (larger than 40 mm anteroposterior diameter)
Study endpoints:
AAA expansion and rates of repair or rupture
Copyright American Society of Nuclear Cardiology

## Slide 4
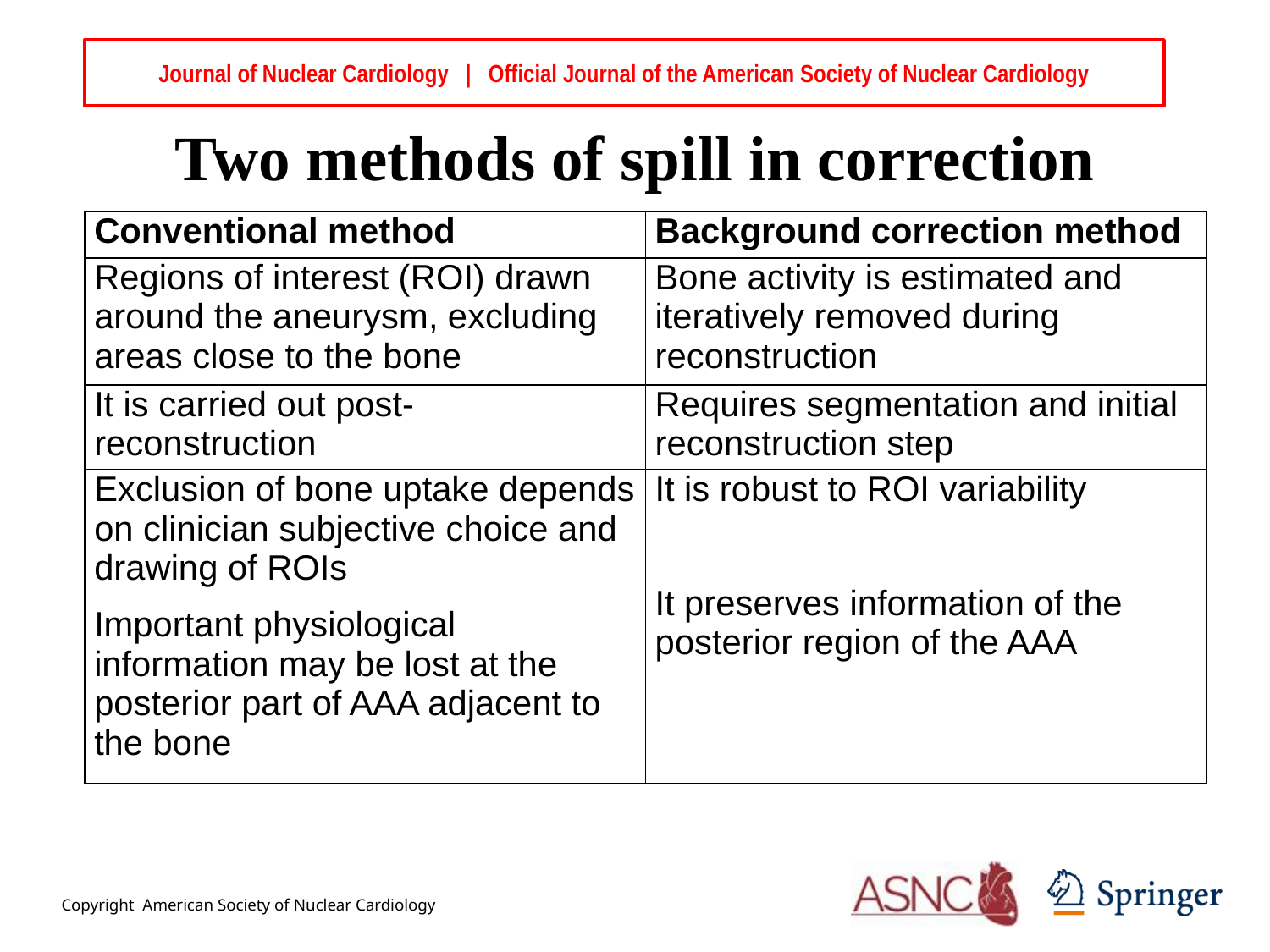

Journal of Nuclear Cardiology | Official Journal of the American Society of Nuclear Cardiology
# Two methods of spill in correction
| Conventional method | Background correction method |
| --- | --- |
| Regions of interest (ROI) drawn around the aneurysm, excluding areas close to the bone | Bone activity is estimated and iteratively removed during reconstruction |
| It is carried out post-reconstruction | Requires segmentation and initial reconstruction step |
| Exclusion of bone uptake depends on clinician subjective choice and drawing of ROIs Important physiological information may be lost at the posterior part of AAA adjacent to the bone | It is robust to ROI variability It preserves information of the posterior region of the AAA |
Copyright American Society of Nuclear Cardiology

## Slide 5
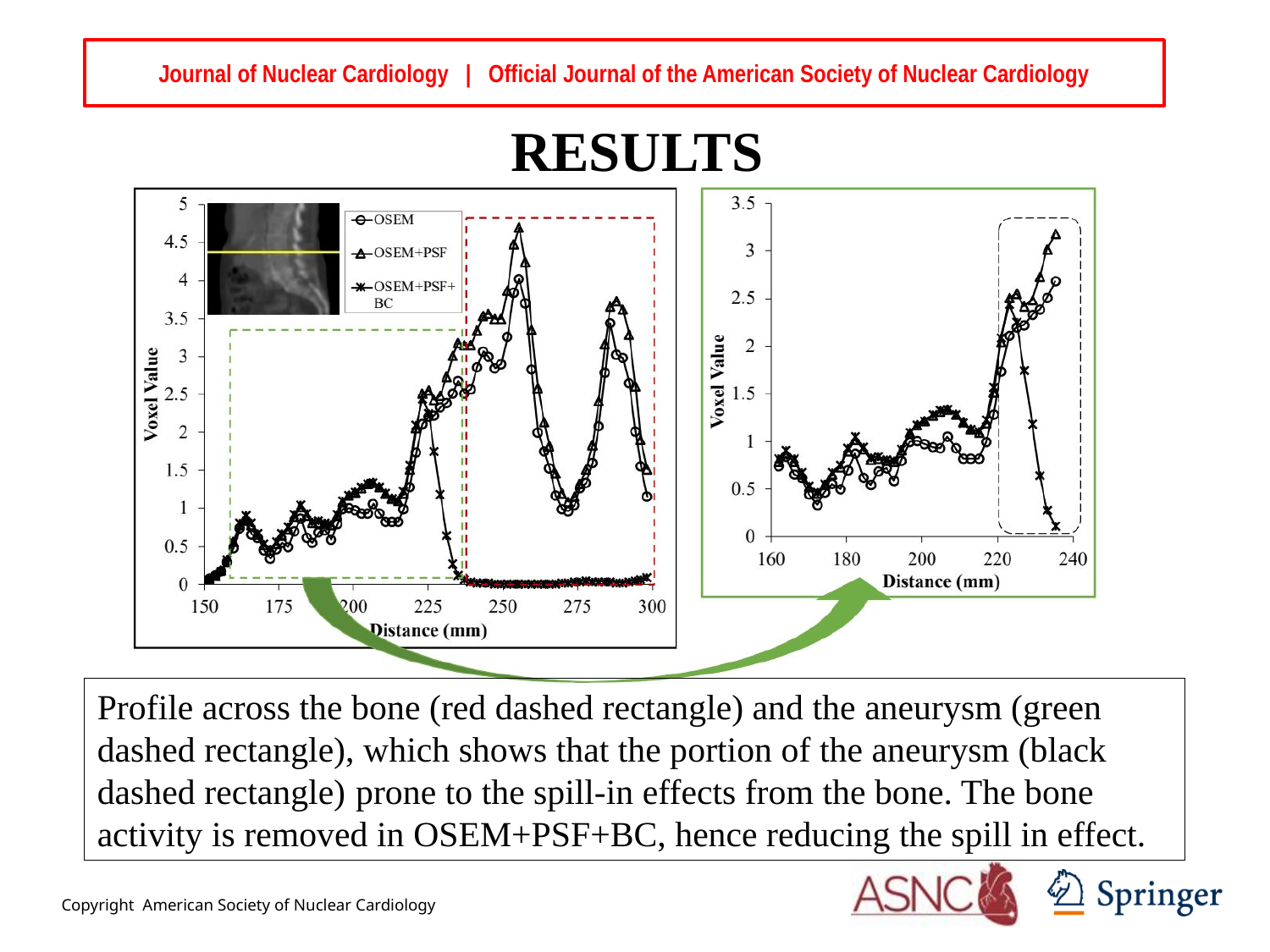

Journal of Nuclear Cardiology | Official Journal of the American Society of Nuclear Cardiology
# RESULTS
Profile across the bone (red dashed rectangle) and the aneurysm (green dashed rectangle), which shows that the portion of the aneurysm (black dashed rectangle) prone to the spill-in effects from the bone. The bone activity is removed in OSEM+PSF+BC, hence reducing the spill in effect.
Copyright American Society of Nuclear Cardiology

## Slide 6
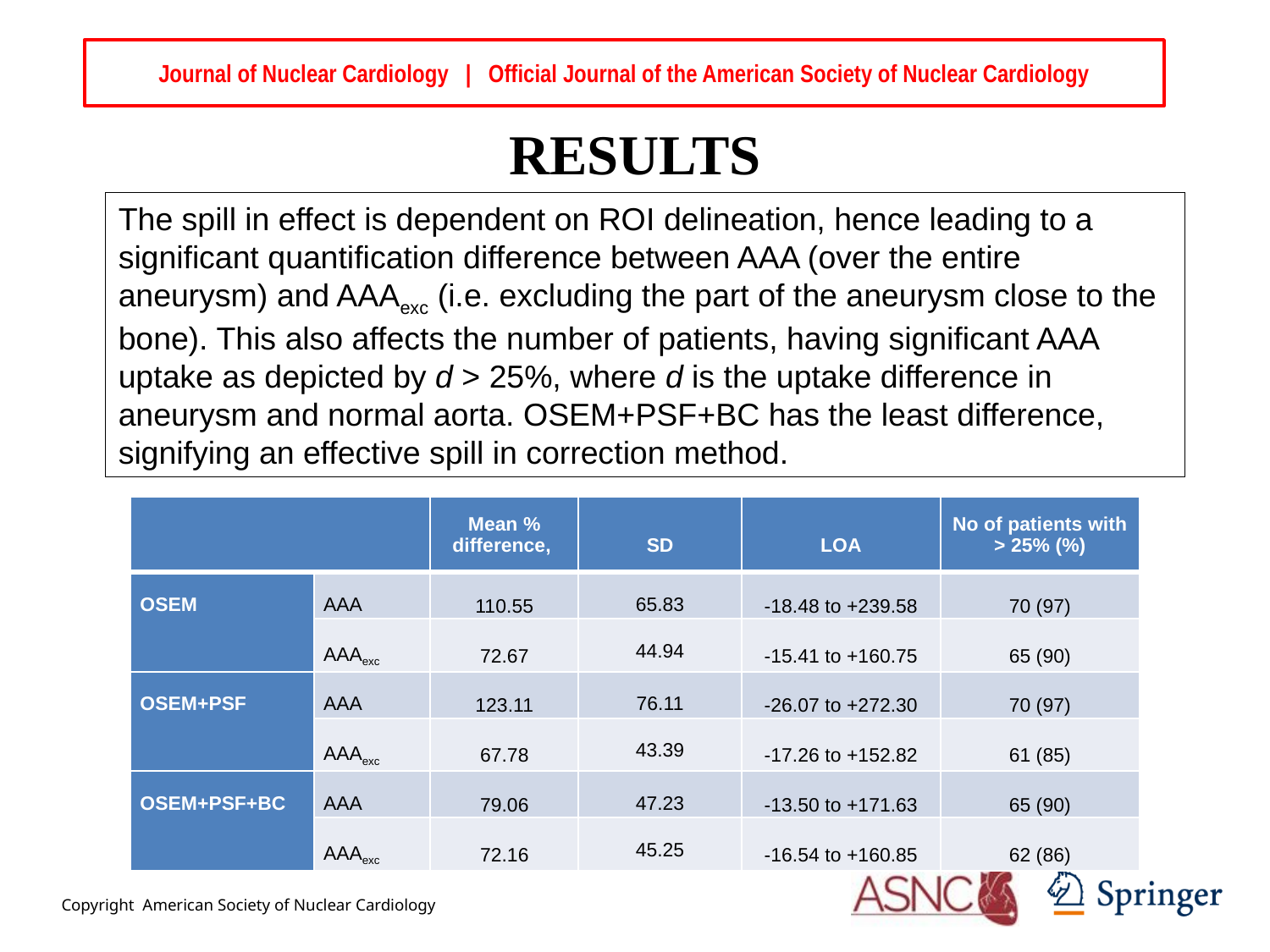

Journal of Nuclear Cardiology | Official Journal of the American Society of Nuclear Cardiology
# RESULTS
The spill in effect is dependent on ROI delineation, hence leading to a significant quantification difference between AAA (over the entire aneurysm) and AAAexc (i.e. excluding the part of the aneurysm close to the bone). This also affects the number of patients, having significant AAA uptake as depicted by d > 25%, where d is the uptake difference in aneurysm and normal aorta. OSEM+PSF+BC has the least difference, signifying an effective spill in correction method.
Copyright American Society of Nuclear Cardiology

## Slide 7
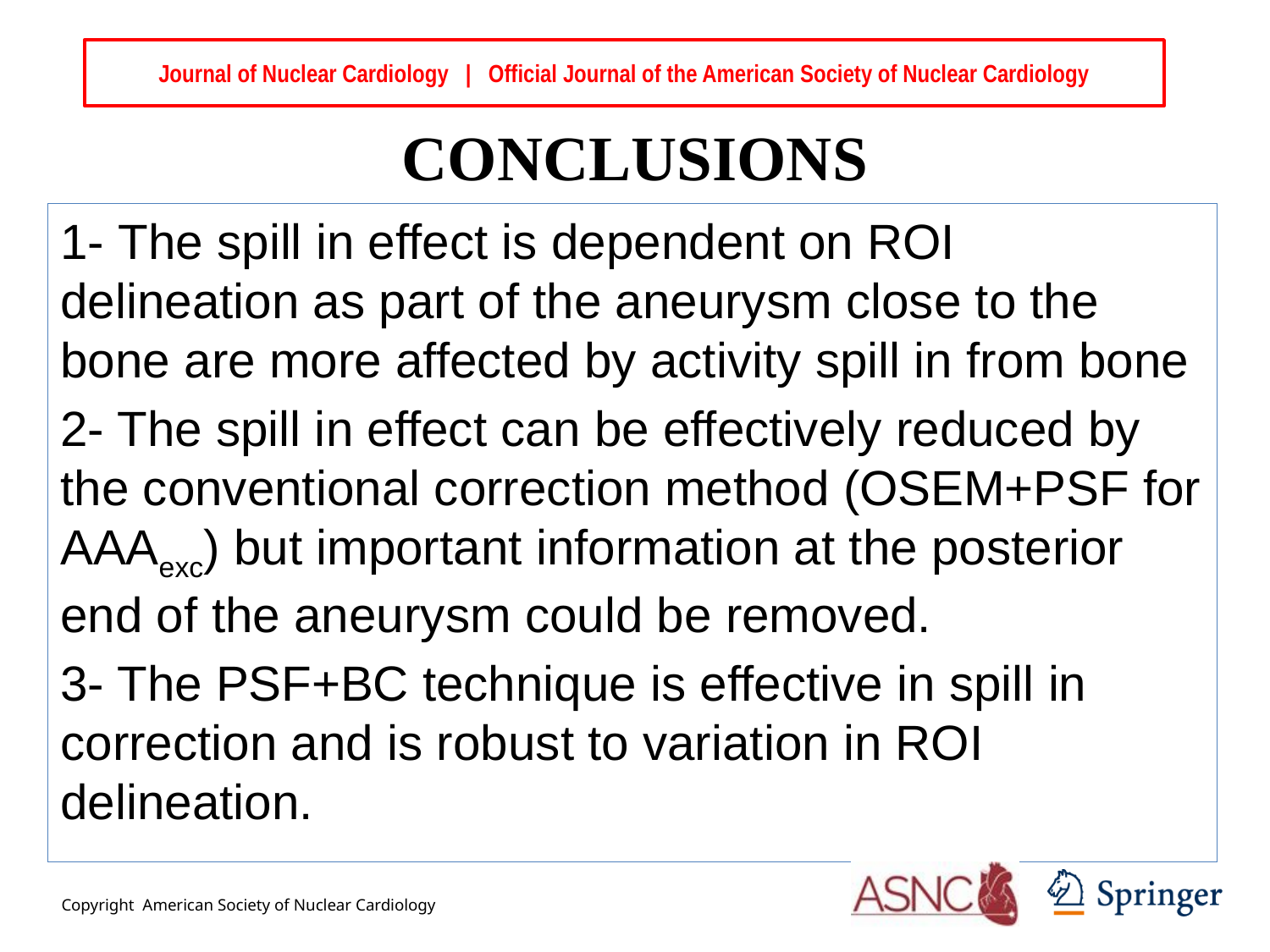

Journal of Nuclear Cardiology | Official Journal of the American Society of Nuclear Cardiology
# CONCLUSIONS
1- The spill in effect is dependent on ROI delineation as part of the aneurysm close to the bone are more affected by activity spill in from bone
2- The spill in effect can be effectively reduced by the conventional correction method (OSEM+PSF for AAAexc) but important information at the posterior end of the aneurysm could be removed.
3- The PSF+BC technique is effective in spill in correction and is robust to variation in ROI delineation.
Copyright American Society of Nuclear Cardiology
